# ­Pharmacokinetics of antimalarial drugs used to treat uncomplicated malaria in breastfeeding mother-infant pairs: An observational pharmacokinetic study

**DOI:** 10.12688/wellcomeopenres.18512.2

**Published:** 2023-08-10

**Authors:** Ritah Nakijoba, Aida Nakayiwa Kawuma, Francis Williams Ojara, Jovia C. Tabwenda, Jacqueline Kyeyune, Christine Turyahabwe, Simon Peter Asiimwe, Johnson Magoola, Clifford George Banda, Barbara Castelnuovo, Allan Buzibye, Catriona Waitt

**Affiliations:** 1 Infectious Diseases Institute, Makerere University College of Health Sciences, Kampala, 256, Uganda; 2 Department of Pharmacology and Therapeutics, Gulu University, Gulu, 256, Uganda; 3 Malawi-Liverpool-Wellcome Clinical Research Programme, Blantyre, 265, Malawi; 4 Department of Pharmacology and Therapeutics, University of Liverpool, Liverpool, L69 7BE, UK

**Keywords:** malaria, breastmilk, pharmacokinetics, Africa, lactation

## Abstract

**Background: **Data surrounding the exposure of the breastfed infant to drugs and any associated risks are sparse. Drugs usually are transferred to milk in small quantities, and many have been used without obviously noticeable infant toxicity for many years – this lack of a ‘safety signal’ has further reduced the interest in studying mother-to-infant transfer of the drugs. In sub-Saharan Africa, pregnant women are at risk of *Plasmodium falciparum* infection, and one in four women have evidence of placental infection at the time of delivery. Artemisinin-based combination therapies (ACTs), primarily artemether-lumefantrine (AL), are the current first-line treatment for uncomplicated *Plasmodium falciparum* malaria, with the same dosing recommendations in breastfeeding women as those in the adult population. Dihydroartemisinin-piperaquine (DP) is routinely used as an alternative to AL in Uganda. However, lactation pharmacokinetics (PK) of ACTs are unknown. Pharmacokinetic characterization of anti-malarial transfer to breast milk and breastfed infants is crucial in understanding the potential consequences to the infant, in terms of therapeutic- and prophylactic effects as well as potential toxicity.

**Methods:** This observational study will enroll 30 mother-infant pairs, and aims to characterize the breastmilk transfer of antimalarial medications (AL and DP) to infants when these ACTs are administered to mothers as part of treatment for uncomplicated malaria. In addition, we will assess the mental health of the breastfeeding mothers enrolled as well as the well-being of their children.

PK samples of maternal blood, breastmilk and breastfeeding infant’s blood will be obtained at specific times points. Pharmacokinetic data will be analyzed using a population pharmacokinetic approach.

**Conclusions: **We anticipate that findings from this research will guide to develop a PK model describing lumefantrine and piperaquine disposition and will provide a framework to foster other lactation pharmacokinetic studies in different disease areas.

## Introduction

Worldwide, around 50% of women take medication during breastfeeding ^1^. Data surrounding the exposure of the breastfed infant to drugs and any associated risks are sparse ^2^. Despite long-standing recommendations from the US Food and Drug Administration (FDA) for lactation studies to be performed close to licensing of drugs anticipated to be widely used in women of childbearing age ^3^, such studies are rarely undertaken.

In sub–Saharan Africa, 25 million pregnant women are at risk of *Plasmodium falciparum* infection every year, and one in four women have evidence of placental infection at the time of delivery ^4^. Artemisinin-based combination therapies (ACTs), primarily artemether-lumefantrine (AL), are the current first-line treatment for uncomplicated *plasmodium falciparum* malaria, with the same dosing recommendations in breastfeeding women as those in the adult population. However, lactation pharmacokinetics (PK) of ACTs are unknown ^5^. In the case of AL, whilst artemether has a very short plasma half-life of approximately two hours, lumefantrine has a plasma half-life of six days and is lipophilic. Therefore, accumulation in breastmilk is likely. Desbutyl-lumefantrine, an active metabolite of lumefantrine, has a significantly higher (>5-times) antimalaria potency than the parent drug. However, the significantly higher mean *in vivo* ratio of lumefantrine to desbutyl-lumefantrine suggests that lumefantrine is the major driver of the antimalarial effect making it the focus of our current analysis ^6^. In Uganda, infant concentrations of lumefantrine, 4 days after completing treatment, predicted relapse ^7^; it is unknown how concentrations in the breastfed infant relate to therapeutic targets. Additionally, there is insufficient information on the safety of infant exposure to breastmilk AL. Out of 6000 children receiving AL, 4726 adverse events were reported, mainly gastro-intestinal (GI) disturbance and cough ^8^ with unknown consequences of potential breastmilk exposure. However, the authors concluded that Artemether-lumefantrine combination is as safe as ASAQ and DP for use in children.

In several regions of the world, resistance to ACTs is increasingly detected ^9^. Dihydroartemisinin-piperaquine (DP) has been evaluated for use in populations including pregnant women ^10^ and is under consideration as an alternative first-line treatment by several countries. In malaria-endemic settings, DP is already being used as an alternative first line for malaria treatment. Like lumefantrine, piperaquine has a long half-life of around 30 days ^11, 12^ and is lipophilic. Therefore, it has the potential to accumulate in breast milk. However, there is limited evidence on what concentrations of piperaquine transfer to breast milk and whether these concentrations are related to efficacious targets or are sub-therapeutic. Like lumefantrine, the major metabolite of piperaquine also demonstrates anti-plasmodial activity. The longer plasma half-life of piperaquine suggests higher exposure compared to the metabolite and hence piperaquine is the focus of this evaluation ^13^.

Drugs are transferred to milk in small quantities, and many have been used without obviously noticeable infant toxicity for many years – this lack of a ‘safety signal’ has further reduced the interest in studying mother-to-infant transfer of the drugs. However, PK characterization of anti-malarial transfer to breast milk and breast-fed infants is crucial in understanding the potential consequences to the infant, in terms of therapeutic- and prophylactic effects as well as potential toxicity. Whilst data on antimalarial excretion/distribution into breast milk is limited, information on clinically relevant infant exposure to antimalarials is even more limited. This is an important knowledge gap both for effectiveness and safety, and because sub-therapeutic concentrations could select for resistance if an infant develops clinical malaria whilst the mother is on treatment, which is a realistic possibility in malaria-endemic regions.

Also, breastfeeding women face mental health challenges whilst taking medications, including postpartum depression, regularly comorbid with anxiety. Approximately 30% of women experience postpartum depression and continue to be depressed up to two years postpartum, while 50% of these women continue to have major depression throughout, and in some cases beyond, the first year postpartum ^14^; thus it is important to assess the psychological status of a breastfeeding woman.

In the present observational study, we aim to characterise the breastmilk transfer of antimalarial medications (AL and DP) to infants when these ACTs are administered to mothers as part of treatment for uncomplicated malaria. In addition, we will assess the mental health of the breastfeeding mothers enrolled, as well as the well-being of their children.

## Protocol

### Disease setting/patient population

Breastfeeding women and breastfed infants, including those living with HIV, and treated for malaria will be recruited prospectively from the Infectious Diseases Institute (IDI) clinic and IDI Kampala City Council Authority (KCCA) affiliated clinics. KCCA clinics are government-funded health facilities providing antenatal and curative services to the population in and around Kampala, including pregnant and breastfeeding women infected with malaria. These clinics were chosen because of their proximity to IDI, availability of laboratory services to confirm malaria infection and the small sample size we require. We anticipate that 5–10% of the women we enroll will be living with HIV. This is based on the prevalence of HIV among pregnant women in Uganda. Drug-drug interactions between antiretroviral therapy (ART) and antimalarials could affect the PK exposure of antimalarials, although a healthy volunteer study conducted at IDI has demonstrated that the first-line dolutegravir-containing antiretroviral regimen can safely be given with artemether-lumefantrine and artesunate-amodiaquine ^15^, there were no significant differences in PK parameters of Artemether with or without dolutegravir containing antiretroviral drugs. Data on concomitant medication will be recorded and included as a covariate in the pharmacokinetic analysis.

## Study objectives

### Primary

1. To define the transfer of lumefantrine and its active metabolite, desbutyl-lumefantrine from mother to breastfed infant.
2. To determine the clearance, area under the concentration-time curve (AUC), and volume of distribution of lumefantrine and desbutyl-lumefantrine.

### Secondary

1. To describe covariates influencing drug exposure in maternal plasma, breast milk and infant plasma.
2. To develop a population pharmacokinetic model including the breast and the infant as compartments, which will enable optimal use of sparse data for optimal dose predictions in future studies as well as simulations of different doses or combinations.
3. To assess depression and anxiety levels among breastfeeding mothers on first-line antimalarial drugs.
4. To assess beliefs about medicines in breastfeeding mothers receiving antimalarial treatment.
5. To determine the clearance, AUC, and volume of distribution of piperaquine.

## Study endpoints

### Primary endpoints

1. Concentrations of lumefantrine and desbutyl-lumefantrine in maternal plasma and breastmilk at 0, 2, 4, 6, 8 and, in some cases, 24 hours, 3, 5, 7, and up to 14-days post-dose
2. Concentrations of lumefantrine and desbutyl-lumefantrine drugs in infant plasma at maternal pre-dose, and up to 8 hours post maternal dose. In some cases, blood concentrations at 5, 7, up to 14-days post maternal dose.
3. AUC of lumefantrine and desbutyl-lumefantrine in maternal plasma and breastmilk
4. Breast milk to maternal plasma (M:P) ratio of lumefantrine and desbutyl-lumefantrine.

### Secondary endpoints

1. Maximum concentration (Cmax) and time to maximum concentration (Tmax) of piperaquine in maternal plasma and breastmilk
2. Infant development (using gross motor development score)
3. Depression and anxiety assessments for breastfeeding mothers
4. Beliefs about medicines in breastfeeding mothers receiving malaria treatment

## Study design

Lactating and breastfeeding women requiring treatment for uncomplicated malaria using either artemether-lumefantrine or dihydroartemisinin-piperaquine will be identified and invited for sampling. Artemether-lumefantrine comprises six doses of medication, with the initial two doses given 8 hours apart on day one, and dosing 12-hourly on day two and day three. Intensive pharmacokinetic sampling will be undertaken after dose five, as indicated in the schema under Table 1 and Table 2. Plasma and breastmilk samples will be obtained pre-dose and at two, four, six, and eight hours after the dose. In addition, sparse sampling will be undertaken on either of these occasions; at pre-dose and between one to six hours after the first dose; a trough (pre-dose) sample after dose three or four and lastly at five, seven, and up to 14-days after the first dose. A heel prick sample will also be obtained from the breastfed infants at maternal trough (before maternal dose) and at a random time point (once per infant) over the eight-hour pharmacokinetic sampling visit to characterize concentrations of these drugs over an eight-hour dosing interval. In addition, a single heel prick sample will be obtained from the infant whenever the mother returns after treatment for the late sampling time points (five, seven, and 14 days post the first dose). Due to the long plasma half-life of lumefantrine (approximately six days), sampling will be performed up to day 14 to characterize the terminal elimination of the drug. We will quantify concentrations of total plasma and breastmilk lumefantrine and desbutyl-lumefantrine.

**Table 1.** Schema illustrating overall study design for mothers receiving artemether-lumefantrine (AL) and dihydroartemesinin-piperaquine (DP).

		Days relative to malaria diagnosis				Days relative to first dose of antimalarials					
		1	2	3	4	5	7 (+/- 1)	14 (+/- 2)	21 (+/- 3)	28 (+/- 3)	35 (+/-3)
**AL**	**Study Procedure**										
	Diagnosis of malaria at clinic	X									
	Screening and enrolment	X									
	Dispensing of antimalarials from clinic	X									
	Pre-dose blood and breastmilk	X									
	Observe first dose	Dose 1									
	Post-dose samples (1, 2, 4, 6 h)	X									
	Advice to take Dose 2 at 8 pm	Dose 2									
	Verify dose taken at home 8 am		Dose 3								
	Self-record dose taken 8 pm		Dose 4								
	Pre-dose blood, breastmilk, and infant blood 8 am			X							
	Directly observed dose, 8 am			Dose 5							
	Intensive PK day (see separate schema)			X							
	Self-record dose taken 8 pm			Dose 6							
	12 h post final dose sampling				X						
	Single samples of blood, breastmilk, infant blood					X	X	X			
	End of study									X	
**DP**	Diagnosis of malaria at clinic	X									
	Screening and enrolment	X									
	Dispensing of antimalarials from clinic	X									
	Pre-dose blood and breastmilk	X									
	Observe first dose	Dose 1									
	Post-dose samples (1, 2, 4, 6 h)	X									
	Advice to take Dose 2 at 8 am next day	X									
	Verify dose taken at home 8 am		Dose 2								
	Pre-dose blood, breastmilk, and infant blood 8 am			X							
	Directly observed dose, 8 am			Dose 3							
	Intensive PK Day (see separate schema)			X							
	Single samples of blood, breastmilk, infant blood				X		X	X	X	X	X
	End of study										X

**Table 2.** Schema of study procedures on intensive lactation pharmacokinetics (PK) day (after dose 5 of artemether-lumefantrine (AL) or dose 3 of dihydroartemesinin-piperaquine (DP)).

		Study Period (8h) ^, time relative to maternal dose						
Subject	Study Procedure	On arrival	0h	2h	4h	6h	8h	24h ^
Mother	Confirm willingness to proceed	x						
	Clinical assessment	x						
	BMI	x						
	Blood for creatinine and albumin			x				
	Blood PK sample	x		x	x	x	x	x
	Breastmilk PK sample	x		x	x	x	x	x
	Standard meal		x			x		
	Observed dosing		x					
	PHQ9, GAD7 and BMQ questionnaires				x ~			
Infant	Clinical assessment	x						
	Weight	x						
	PK sample	x				X *		

*Time of second infant DBS to be recorded, between 3 and 8h ^Due to logistic constraints, the 24 hours may not be collected in every participant ~can be any time during PK day

**Figure 1.**
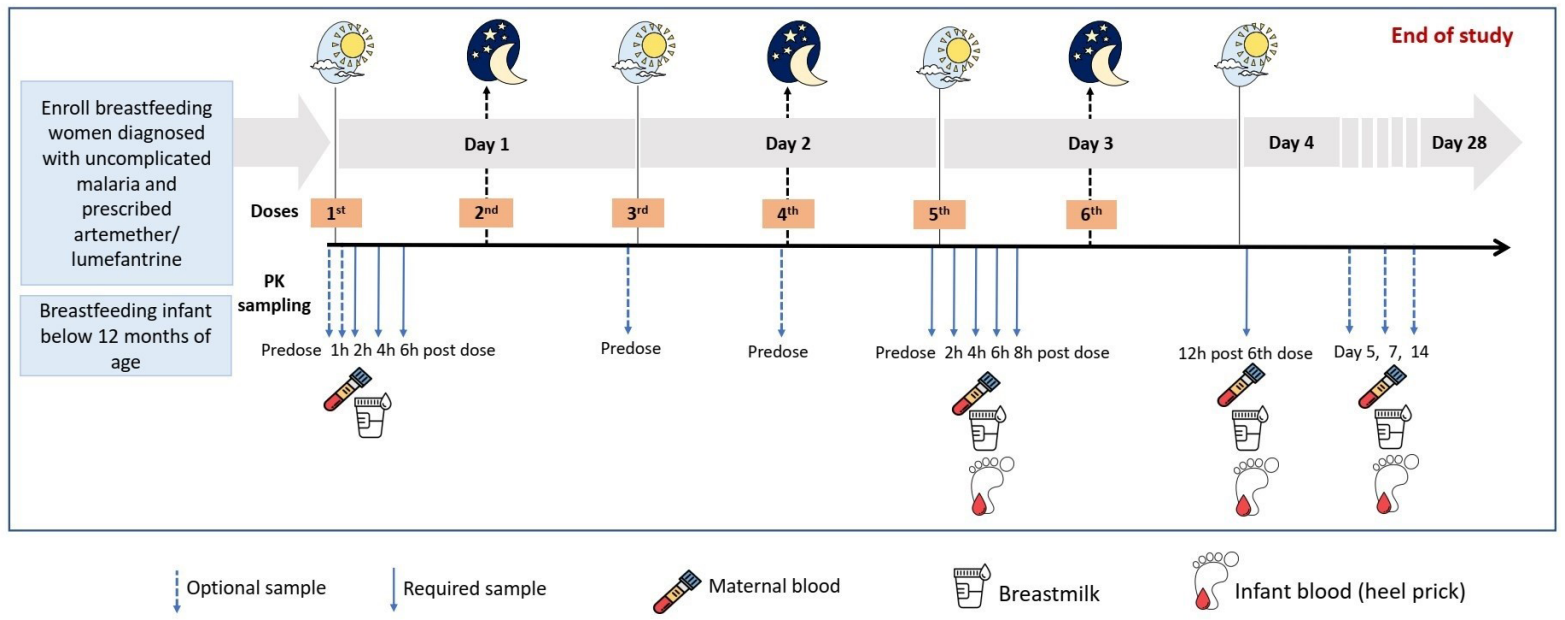
Study design and sampling schedule.

Whilst not yet first line in Uganda, if a breastfeeding mother receiving dihydroartemisinin-piperaquine is identified, she shall be invited for intensive pharmacokinetic sampling as an exploratory endpoint. Study procedures will be the same as for artemether-lumefantrine except that an additional sparse sample may be taken on days 21, 28 and 35 specifically to characterize the elimination given the substantially long plasma half-life of approximately 30 days. The schema in Figure 1 summarizes the study design.

In Uganda, up to 25% of women have children before the age of 18 years. Current guidelines from the Uganda National Council of Science and Technology (UNCST), a body responsible for overseeing the implementation of research in the country, consider adolescent mothers aged 14–17 as emancipated minors. As such, they can provide their consent to participate in research (rather than parental consent followed by child assent). These younger aged mothers will specifically be targeted for inclusion in the study to be representative of this highly marginalized and vulnerable group. This is suited to the objectives of the related Wellcome-funded public engagement enrichment award, ATtaining EQUity of Access TO Research (At The EQUATOR) whose overall objective is to ensure that underrepresented populations are involved in research.

## Participant selection

### Inclusion criteria

Participants must meet all the following inclusion criteria to be eligible for enrollment in the study.

1. A personally signed and dated informed consent document indicating that the participant has been informed of all pertinent aspects of the study.

2. Participants who are willing and able to comply with scheduled visits, treatment plans, laboratory tests, and other study procedures.

3. A woman is aged 18 years or older, or an emancipated minor aged 14–17 according to the Uganda National Council of Science and Technology Uganda National Council of Science and Technology (UNCST) guidelines ^16^.

4. Receiving treatment for uncomplicated malaria

5. Breastfeeding at enrolment

### Exclusion criteria

Participants presenting with any of the following will not be included in the study:

1. Severe maternal or infant illness, which in the opinion of the patient’s clinician would interfere with her participation in the study.

2. Breastfed infant is aged over 12 months. Time-dependent changes in the quality of breast milk and infant feeding practices may affect mother-to-infant transfer of drugs, hence a cut-off period of 12 months is set to control for possible heterogeneity in exposure.

3. Partner objection to participating in the study

## Treatments of participants, drug storage and drug accountability

This is an observational study; hence no changes will be made to the administered medication. All women (and infants) will continue to receive their malaria treatment as prescribed by the physician where they receive clinical care. Drugs will be stored and dispensed from the relevant clinic pharmacy, with no special procedures relating to this observational protocol. At every study visit, women will be asked about concomitant medication and the results noted on a specific case report form (CRF).

## Study procedures

### Informed consent

Women will be identified as they attend the clinic for malaria treatment at the IDI and KCCA clinics in Uganda. Should a woman express willingness to participate, once her eligibility for enrolment in the study has been determined, informed consent will be obtained.

Each potentially eligible participant must sign an informed consent form before the conduct of any screening procedures. Participants will be allowed to ask any questions regarding the study at this stage. Screening evaluations will be used to determine the eligibility of each candidate for study enrolment.

If the participant is unable to read and/or write, an impartial witness will be present during the informed consent discussion. The witness should be able to read the consent form and participant information leaflet in the participant’s chosen language. After the written, informed consent form is read and explained to the participant, and after they have orally consented to their participation in the study and have either signed the consent form or provided their fingerprint, the witness will sign and personally date the consent form. By signing the consent form, the witness attests that the information in the consent form and any other written information was accurately explained to, and apparently understood by, the participant and that informed consent was freely given by the participant.

### Screening

At the screening visit, the participant will be evaluated against the eligibility criteria. If she is eligible, arrangements will be made for her to come to the IDI Clinic before taking her dose 1 of AL. For participants who have received intravenous artesunate before AL, enrolment will be for AL treatment and sampling. If this is logistically impractical for a breastfeeding mother-infant pair, she can be invited to attend for sampling before intake of dose 3, and in all cases for intensive sampling after dose 5. In the Uganda public health sector, intravenous artesunate is commonly used as empirical treatment (without the confirmation of complicated malaria) and therefore the use of intravenous artesunate prior to oral AL will not preclude a potential participant from being enrolled in the study provided there are no clinical features of severe malaria.

The study is being performed because there is no clear data that describe how much drug transfers from mother to breastfed infant. As this is an observational study, it would already have been decided by the treating clinician that the mother requires the medication for her health and that the benefits outweigh the risks. Exclusive breastfeeding complies with WHO guidelines. There are no specific harms relating to the malaria treatment about which we should specifically inform the mother prior to participation in the study, but rather the study aims to increase understanding.

### Pharmacokinetic sampling procedures

***Day 1 (sampling after dose 1)***

If the mother can be transferred to IDI for dose one, a blood and breastmilk sample will be taken before directly observed dosing. Depending on the time of day, further paired blood and milk samples will be taken at one, two, four- and six-hours post-dose. Mother will continue breastfeeding their infants normally. If she is unable to attend IDI on day one of treatment, arrangements will be made for her to attend in the morning of day two, for paired sampling before intake of dose three.

***Day 3 (sampling after dose 5)***

On arrival, an intravenous cannula will be inserted into her antecubital fossa, and blood and breastmilk samples will be taken for trough drug measurement. After a standardized high-fat breakfast she will be administered her medication. Blood and breastmilk samples will be collected at two-, four-, six-, and eight-hours post-dose. To evaluate infant exposure to the drug through breastmilk, an infant sample will be taken before maternal dosing (to provide estimated infant trough concentration) and at a time point between three and eight hours after maternal dosing (to estimate maximum infant concentration); this approach to infant sampling has been validated in other lactation pharmacokinetic studies in Uganda. For mothers on dihydroartemisinin/piperaquine, day 3 sampling will be performed after the third and last dose of treatment and a minimal-fat containing standardised breakfast will be provided.

Maternal albumin and creatinine will be sampled as these may be important covariates that influence pharmacokinetic parameters. Maternal questionnaires will be completed on each visit to assess depression and anxiety; these include the Generalised Anxiety Disorder questionnaire (GAD-7), participants with abnormal mental health screenings will be referred to the institution mental health clinic for further review. The Patient Health Questionnaire (PHQ-9), and the Beliefs about Medicines Questionnaire (BMQ). Infant clinical assessment will include the use of the gross motor development (GMD) checklist, see Appendix.

***Later time points***

As detailed above, lumefantrine and piperaquine both have long half-lives, and to fully understand how long the drugs are measurable in the body, and how long they take to be cleared from the maternal blood, breastmilk and infant plasma, the mother and infant will be invited for further study visits on days five, seven, and up to 14 days after the first dose of treatment. For mothers on DP, additional samples may be taken on day 21, 28 and 35. On these occasions, a single sample of maternal blood, breastmilk and infant blood will be collected.

A study of the population pharmacokinetic of lumefantrine and its metabolite in children with uncomplicated malaria reported a median (interquartile ranges) DBL-to-lumefantrine-drug ratio of 1.13% (0.93-1.55) % calculated as AUC _metabolite_/AUC _parent_ (Salman *et al*.). Given that the terminal elimination half-life of DBL is reported to be longer than that of lumefantrine, we expect the DBL/lumefantrine ratio to increase with time.

## End of malaria treatment outcomes

The endpoints of this study relate to the concentrations of antimalarial drugs present in maternal blood, breastmilk, and infant blood. The study is not powered for antimalarial efficacy, and therefore formal assessment of parasitological clearance is not required. The participants will be followed up until 30-40 days after completion of antimalarial therapy, and if recurrent symptoms occur, management will be as clinically indicated. Details regarding further clinical investigations and management required by either mother or infant during the follow-up period will be recorded on the CRF.

## Participant withdrawal

Participants may withdraw from the study at any time at their own request, or they may be withdrawn at any time at the discretion of the investigator or sponsor for safety or behavioral reasons, or the inability of the participant to comply with the protocol required schedule of study visits or procedures.

If a participant does not return for a scheduled visit, every effort will be made to contact the participant. At enrolment, participants will have been asked to provide contact numbers and directions to their home. Initial attempts will be made to speak to the participant by telephone, ascertaining the reasons for not attending the clinic. In any circumstance, every effort will be made to document participant outcome, if possible. The investigator will inquire about the reason for withdrawal and follow-up with the participant regarding any unresolved adverse events (AEs).

If the participant withdraws from the study, and withdraws consent for disclosure of future information, no further evaluations will be performed, and no additional data will be collected.

## Assessments

### Safety

Given that this is an observational study, we do not have concerns about the risk-benefit ratio to the individual participant. However, we will appropriately record and respond to adverse events related to the study procedures. These events might include adverse events relating to sampling of maternal blood, breastmilk, or infant blood; these will be detailed on the case report form, and the participant followed until the event has resolved. Any events that do occur will be listed and analysed according to DAIDS criteria. Given that this is not a clinical trial, and the medication used is not an investigational product, there is no formal requirement for notification of adverse events to the regulatory authorities.

The potential risk to healthcare workers is not considered higher than routine clinical work in this environment, where incidence and prevalence of HIV and TB are both high. Appropriate universal precautions will be taken.

### Pharmacokinetic assessments

***Blood for pharmacokinetic analysis***

All efforts will be made to obtain the pharmacokinetic samples at the scheduled nominal time relative to dosing. If a scheduled blood sample collection cannot be completed for any reason, the missed sample time may be re-scheduled with agreement of clinical investigators

***Sample handling***

Maternal plasma breastmilk and infant plasma will initially be processed and stored in a -80°Celsius freezer at IDI core laboratory. Drug concentrations will be determined in plasma and total breastmilk using liquid chromatography-tandem mass spectrometry at the IDI laboratory

***Bioanalysis***

This protocol aims to contribute to bioanalytical capacity in Uganda and all methods will be developed and performed at IDI. The IDI bioanalytical laboratory is a GCLP certified laboratory that undertakes drug concentration measurement in plasma. It possesses vast experience in method development for several priority drugs including antimalarials, anti-tuberculosis and antiretroviral drugs. It currently houses a liquid chromatography-tandem mass spectrometry (LC-MS/MS) workstation with assays developed and validated according to United States Food and Drug Administration (FDA) standards. An LC-MS/MS method exists for the determination of lumefantrine in plasma. This will be modified to include piperaquine and further optimized for breast milk. Therefore, running the samples in-house at IDI will offer an opportunity for further training of laboratory technologists and provide international exposure for the IDI laboratory and its capabilities. This also will increase the precedent for performing bioanalysis within the country wherever possible. No shipment is therefore necessary. However, the study has a collaboration between IDI, University of Liverpool and University of Cape Town, and that in both partner sites there are GCLP accredited laboratories. Liverpool has significant strength in antiretroviral drugs, and UCT in antituberculotic drugs.

The lab has an EQA system with the Netherlands laboratory, and the study has a collaborative partnership with the UCT laboratory whereby the laboratory staff receive regular mentorship and technical guidance remotely. Also, the IDI lab team have strong working relationships with the lab teams at both of these partner organizations.

***Validation of breastmilk assay/ donor breastmilk***

The method for the determination of lumefantrine and piperaquine will be validated both in plasma and breast milk according to the FDA guidelines for bioanalytical method development ^17^. It is essential that the standards made to form the standard curve and the different levels of quality control (QC) are spiked into the matrix (plasma, milk, urine etc.) which will be used for the clinical samples. Given there is not yet a fully functioning donor human milk bank in Uganda, we will invite breastfeeding women who are not taking any medication to donate a small amount of breast milk for scientific reasons. These are likely to be staff and students working within IDI, and this approach is frequently used in other centres for the donation of ‘blank’ blood for assay validation.

## Data analysis/statistical methods

### Sample size determination

This study is exploratory, as no prior study has characterized the exposure of these drugs in maternal plasma, breastmilk, and infant plasma. There are no prior data upon which to build a sample size calculation, and there is no comparison between groups which require statistical analysis with a pre-specified certainty.

Early studies in rats report a lumefantrine milk to plasma ratio of 1.4 ^18^. As such, we expect that lumefantrine accumulates in human breastmilk. Therefore, the approach we have adopted is to undertake a study design that allows us to adequately describe the pharmacokinetics of the antimalarial drugs in plasma. Plasma kinetics will then be linked to drug concentrations observed in the breastmilk and infant.

To evaluate the robustness of our approach, we undertook a stochastic simulation and estimation exercise using a previously published model describing lumefantrine population pharmacokinetics ^19^. We simulated lumefantrine concentrations using the study design and sampling schedule outlined in section 3 for an average nursing mother weighing 70 kg on standard adult AL treatment (6 doses of 80 mg/480 mg).

Our simulations show that with the proposed sampling schedule (intense sampling after 5 ^th^ dose and sparse sampling after 1 ^st^ and 6 ^th^ dose), we can estimate lumefantrine pharmacokinetic parameters with good precision (relative standard error of less than 30%) for all parameters including absorption rate constant, clearance, and volume of distribution. In addition, on average, we should expect lumefantrine plasma concentrations of 87.6, 5.28 and 0.492 ng/mL on day 8, 12 and 16 post the final dose, respectively. Given our validated lumefantrine assay has a lower limit of quantification of 200 µg/L (200 ng/mL), we anticipate that we will not be able to reliably quantify lumefantrine plasma concentrations after about 10 days. Based on these simulation results we expect to be able to adequately describe lumefantrine plasma disposition and in turn, link it to breastmilk disposition using for example an effect compartment ^20^.

We will aim to perform an interim analysis after data from about five participants are available to help inform whether our earlier assumptions hold.

For piperaquine, we also undertook a stochastic simulation and estimation exercise using a previously published model in adults, with a standard adult dosing regimen of dihydroartemisinin/piperaquine (120/960 mg) administered once daily (24-hourly) for three days. Our simulations show that we can adequately estimate piperaquine disposition with a design that allows for intense maternal PK sampling after the 1 ^st^ (day 1) and 3 ^rd^ dose (day 3), accompanied by a wash-out phase up to 35 days post the first dose. In the wash-out phase, PK samples will be undertaken on days 7, 14, 21, 28, and day 35.

### Analysis of endpoints

Pharmacokinetic data will be analyzed using a population pharmacokinetic approach to estimate pharmacokinetic parameters and produce modelled fits to exposure data ^21, 22^ Inter-individual variability will be quantified in relation to the covariates.

Non-compartmental methods will be used to assess correlations between maternal breast milk drug concentrations and measures of drug exposure in the infant (e.g., AUC) and pharmacodynamic factors.

***Analysis of primary endpoint***

*Population pharmacokinetic (pop-PK) analysis*

Population pharmacokinetic modelling will be used to characterise the change in drug and metabolite concentrations across time and estimate parameter characterising oral absorption of the drug, distribution of the drug in the body i.e., the volume of distribution (V _d_) and clearance of the drug (CL) and derive the AUC corresponding to different time points, time-to-maximum drug concentration (T _max_) as well as concentration at maximum concentration (C _max_).

Drug concentrations in breastmilk will be incorporated in the maternal population pharmacokinetic models using an effect compartment strategy or other novel strategies, which will allow accurate estimation of the accumulation in breastmilk compared to plasma. Exposure to lumefantrine and piperaquine in breastfed infants will be compared with the range of concentrations achieved in adults given therapeutic doses, assuming a minimal duration of exposure from delivery and a maximal duration from the time the mother started the drug (to account for placental transfer)

*Analysis of secondary endpoints*

The data derived from the generalised anxiety disorder questionnaire (GAD-7), Patient health questionnaire (PHQ-9), the beliefs about medicines questionnaire (BMQ) and the infant gross motor development (GMD) checklist will be analysed together with data from the other protocols within the overarching MILK fellowship (mothers on drug-sensitive TB treatment in Uganda and on drug-resistant TB treatment in South Africa) and a broader analysis of medication use in breastfeeding which will be undertaken as a component of the PhD Scholarship held by Ritah Nakijoba under MILK. This combined analysis will allow the exploration of beliefs and attitudes surrounding a range of medications used in breastfeeding mothers.

*Safety analysis*

Safety events relating to the study procedures will be graded and analyzed.

## Quality control and quality assurance

During study conduct periodic monitoring may be conducted to ensure that the protocol and Good Clinical Practices (GCPs) are being followed. The monitors may review source documents to confirm that the data recorded on CRFs is accurate. Additionally, the study site may be participant to review by the Institutional Review Board (IRB) and/or to inspection by appropriate regulatory authorities.

## Data handling/record retention

### Case report forms (CRF)

A CRF is required and will be completed for each included participant.

The investigator has ultimate responsibility for the collection and reporting of all clinical, safety and laboratory data entered on the CRFs and any other data collection forms (source documents) and ensuring that they are accurate, authentic / original, attributable, complete, consistent, legible, timely (contemporaneous), enduring and available when required. The CRFs must be signed by the investigator or by an authorized staff member to attest that the data contained on the CRFs is true. Any corrections to entries made in the CRFs, source documents must be dated, initialed and explained (if necessary) and should not obscure the original entry.

### Record retention and archiving

To enable evaluations and/or audits, the investigator agrees to keep records, including the identity of all participating patients (sufficient information to link records, e.g., CRFs and clinic records), all original signed informed consent documents, copies of all CRFs, safety reporting forms, source documents, and detailed records of treatment disposition, and adequate documentation of relevant correspondence (e.g., letters, meeting minutes, telephone calls reports).

Investigator records must be kept for as long as required by applicable local regulations (10 years in Uganda). When more than one requirement can be applied, records must be maintained for the longest period provided.

### Confidentiality and insurance

Clinical data will be entered into a study specific database by designated staff on a regular basis from completed Case Record Forms (CRF). CRFs and other source documents will be kept in locked cabinets. Data will be entered on a regular basis to ensure that it is up to date. The database will be entered on a regular basis on a secure PC, as will the pharmacokinetic data that will be received by the laboratories. Access to the database will be given to authorized personnel only (members of the immediate study team) and a log of authorized personnel will be stored in the trial master file. CRF and trial documents will be kept in locked cabinets. No participant identifying information will be disclosed in any publication or at any conference activities arising from the study.

This observational study will be covered under the University of Liverpool’s existing policy with Newline Insurance.

## Ethics

### Institutional Review Board (IRB)

It is the responsibility of the investigator to have prospective approval of the study protocol, protocol amendments, informed consent documents, and other relevant documents from the IRB. All correspondence with the IRB will be retained in the regulatory or trial master file. Copies of IRB approvals will be filed with other study documents. The study was reviewed and approved by the Infectious Diseases Institute (IDI) Regulatory Ethics Committee (REC) REF: 20/2022 on 01August 2022, and the Uganda National Council of Science and Technology (UNCST): HS2392ES.

### Ethical conduct of the study

The study will be conducted in accordance with legal and regulatory requirements, as well as the general principles set forth in the International Ethical Guidelines for Biomedical Research Involving Human Participants and the Declaration of Helsinki.

In addition, the study will be conducted in accordance with the protocol, GCP guidelines, and applicable local regulatory requirements and laws.

### Participant information and consent

All parties will ensure protection of participant personal data and will not include participant names on any forms, reports, publications, or in any other disclosures, except where required by laws. The informed consent document used in this study, and any changes made during the course of the study, must be prospectively approved by the IRB. The investigator, or a person designated by the investigator, will obtain written informed consent from each participant or the participant's legal representative before any study-specific activity is performed. The investigator will retain the original of each participant's signed consent document.

## Definition of end of study

The study will be considered complete when the target number of participants has been enrolled and have completed the study period and the data analysis is complete.

## Publication and dissemination of study results

Study findings will be disseminated to the stakeholders using both traditional approaches through articles published in journals, or via conferences as oral or poster presentations and new media platforms like social media, blogs and digital sites that have broadened opportunities.

Some of the identified stakeholders include the National Malaria Programme in the National Department of Health, Kampala Capital City Authority, IDI internal stakeholders, academic institutions, Civil Society Organizations, community groups, the media and general public.

The public engagement strategy has stipulated the various channels and techniques that will be employed to ensure meaningful two-way dialogue throughout. Below we list some of the activities we will undertake/have undertaken during the course of the study.

### During protocol development

A Community Advisory Board (CAB) meeting was held on 17 ^th^ May 2022 to enable full discussion of this protocol, and community feedback was incorporated into the protocol and ICFs prior to initial ethics submission.

### During study set-up

Early consultation with the CAB to determine which community members to approach for specific dialogue on related matters took place on May 17 ^th^, 2022. The following activities are planned for the period between submission and study commencement:

- Initial community meeting with religious leaders, women’s local representative and community members, involving discussion and use of flyers with pictures and simplified, translated text.
- Identification of key community members who are interested in co-creation of materials and resources.
- Establishment of social media channels relating to study activities.
- Engagement with journalists/ media – identify interested parties.

### At site initiation

We will hold a community question and answer session on site to provide specific information on study recruitment. In addition, we plan to hold radio broadcasts, produce skits relating to study activities and circulate tailor-made brochures and posters with photos and simplified text.

### During study

We will hold community meetings every 1–2 months to give updates on study progress. In addition, we will produce a monthly newsletter and engage actively on different social media platforms including Twitter and LinkedIn

## Discussion and conclusions

This protocol has been designed with the aim to determine the pharmacokinetics of first-line antimalarial drugs in breastmilk and describe their transfer to a breastfed infant. It is regrettable that more than 10 years after the use of these drugs as first-line, there is no information on their distribution to breast milk. Uganda, which is a malaria endemic area with a relatively high fertility rate of 4.7 births per woman, is well placed to answer this question and findings from this research would be relevant and beneficial to the country.

Understanding infant exposure to antimalarials is important to inform on potential risks and benefits to the infant. Is the exposure minimal or substantial enough to warrant further investigation, regarding for example, the potential to predispose an infant to selection for resistance? Increasingly, there are reports of resistance to AL and highlighting the role that suboptimal exposure in infants might play would be instructive.

Characterisation of the dynamic time-course of maternal plasma and breast milk concentrations, as well as infant plasma concentration-time profiles will be facilitated by the serial sampling of maternal plasma, maternal breast milk and infant plasma. Directly measuring drug concentrations in the infant will provide a better understanding of infant exposure and enable informed prediction of infant exposure unlike in most lactation pharmacokinetic studies in which maternal-to-infant transfer of drugs (infant exposure) is estimated from only maternal plasma and breast milk drug concentrations.

We have proposed a model-based population analysis approach for characterisation of changes in drug concentrations across time. Population analysis will enable joint analysis of data from multiple participants, describing both the typical population- and individual-specific profiles. Model-based population analysis offers several advantages over the more commonly used non-compartmental analysis. Firstly, model-based population analysis requires fewer pharmacokinetic samples for efficient pharmacokinetic parameter estimation compared to the more often used non-compartmental analysis and reduces the stress associated with more frequent blood draws in breastfeeding mothers. Secondly, model-based population will also enable efficient evaluation of the impact of different patient- and treatment-related factors on drug concentration-time profiles. Thirdly, population pharmacokinetic model analysis will enable testing different distributional assumptions on the transfer of drugs from mother to the infant to inform on the underlying influential biological mechanism. Lastly, the developed pharmacometric model can be used for simulation-based exploration of the impact of different dosing regimens or different patient factors on drug concentration-time profiles.

For this protocol we will aim to include emancipated minors, mothers between the age of 14–17 years. Ideally, participants under the age of 18 would require their parents’ consent to participate in research. However, the Uganda national council of science and technology stipulates that these emancipated minors can give consent and do not require their parents. Given the unfortunate statistic in Uganda, where approximately 25% of women have children below the age of 18, we are compelled to represent this population in our research. This is in an effort to ensure equity in access to health research. We hope to gain some insight into dynamics surrounding the participation of this group in research.

This protocol has been approved by the Infectious Diseases Institute (IDI) Regulatory Ethics Committee (REC), the University of Liverpool REC and the Uganda National Council of Science and Technology. Details of ethical approval have been reported in the ethics section.

In conclusion, we anticipate that findings from this research will go a long way in ensuring optimal use of first-line antimalarial drugs and will provide a framework to foster other pharmacokinetic lactation studies in different disease areas.

## Study status

The study will start recruitment after the site initiation visit by the IDI internal monitor

## Data Availability

No data are associated with this article.
