# Supplementary material for: ­Pharmacokinetics of antimalarial drugs used to treat uncomplicated malaria in breastfeeding mother-infant pairs: An observational pharmacokinetic study
Source: Wellcome Open Res. 2023 Aug 10;8:12. Originally published 2023 Jan 6. [Version 2] doi: 10.12688/wellcomeopenres.18512.2 (PMC10514676; doi:10.12688/wellcomeopenres.18512.2)
Supplement: Peer review history

# Reviewer report: Reviewer response for version 2

**DOI:** 10.21956/wellcomeopenres.22003.r65000

**Published:** 2023-09-19

**Authors:** Mary Ellen Gilder

**Affiliations:** 1 Shoklo Malaria Research Unit, Mae Sot, Tak, Thailand

I would like to thank the authors for their responses. Though I have continued concerns about potential difficulties interpreting PK values from random breast milk samples, I understand that the researchers face constraints. The argument that foremilk and hindmilk concentrations will vary because mothers will be feeding their infants regularly is only partially convincing. Unless the sample is taken from the most recently fed breast and within a few minutes of feeding, there will be an oversampling of foremilk (which may underestimate drug exposure for lipophilic drugs). Women will naturally express milk from the fuller breast unless instructed otherwise, as it will be much quicker and easier. Measuring the creamatocrit of the thawed samples at the central PK lab could be one simple, inexpensive measure that would not burden staff in busy clinics. This could help shed light on relationships between fat and drug concentrations, and help explain variations in drug concentrations between patients and time points, though it would not compensate for systematic oversampling of foremilk. Regardless, this study will add much needed data on antimalarial drugs in breast milk. I wish the authors luck in this important work!

Is the study design appropriate for the research question?

Yes

Is the rationale for, and objectives of, the study clearly described?

Yes

Are sufficient details of the methods provided to allow replication by others?

Partly

Are the datasets clearly presented in a useable and accessible format?

Not applicable

Reviewer Expertise:

malaria, lactation pharmacokinetics, maternal and child health

I confirm that I have read this submission and believe that I have an appropriate level of expertise to confirm that it is of an acceptable scientific standard.

# Reviewer report: Reviewer response for version 2

**DOI:** 10.21956/wellcomeopenres.22003.r65001

**Published:** 2023-08-24

**Authors:** Andre Daher

**Affiliations:** 1 Oswaldo Cruz Foundation, Rio de Janeiro, Brazil

My comments have been addressed properly. No further comments, Congratulations to the authors.

Is the study design appropriate for the research question?

Yes

Is the rationale for, and objectives of, the study clearly described?

Yes

Are sufficient details of the methods provided to allow replication by others?

Yes

Are the datasets clearly presented in a useable and accessible format?

Not applicable

Reviewer Expertise:

Clinical research, drugs, Malaria, NTD.

I confirm that I have read this submission and believe that I have an appropriate level of expertise to confirm that it is of an acceptable scientific standard.

# Reviewer report: Reviewer response for version 1

**DOI:** 10.21956/wellcomeopenres.20528.r55015

**Published:** 2023-03-21

**Authors:** Mary Ellen Gilder

**Affiliations:** 1 Shoklo Malaria Research Unit, Mae Sot, Tak, Thailand

Pharmacokinetics of drugs used to treat uncomplicated malaria in breastfeeding mother-infant pairs: An observational pharmacokinetic study

 In this manuscript Nakijoba et al propose to study the lactation pharmacokinetics of lumefantrine and, as an exploratory endpoint, piperaquine, essential components of first line antimalarial treatments. They aptly point out that this work is long overdue. Overall, I would like to congratulate the authors on this interesting and important planned work. While I have some comments for consideration, I am encouraged to see that researchers are focusing on the neglected area of lactation pharmacokinetics, particularly for long-acting antimalarial drugs. 

**Major concern:**

 My most significant queries relate to the exact nature of the breast milk sampling.

 What is your plan for breast milk sampling eg. random breast milk sample (not recommended) or complete emptying of both breasts for the duration of the study (recommended by FDA but burdensome and possibly unethical)? Use of a random sample is likely to oversample foremilk, which may have lower drug concentrations due to lower fat content than hind milk, especially considering the lipophilicity of these drugs. Emptying of the most full breast at the PK timepoints and taking an aliquot of this milk after thorough mixing is a practical option which approximates the dynamics of breastfeeding. (Separate testing of foremilk and hindmilk could be done in selected samples if feasible.)

 Do you plan to measure the fat content of the milk samples (eg. by creamatocrit) to help explain fluctuations in drug concentrations?

 If women are fully emptying one or both breasts, what will be done with the remaining milk after the aliquot is taken? If it will be fed back to the infant, what measures are in place to minimize disruption of the breastfeeding relationship (eg. will bottles be used, or will the milk be fed by other methods?) What lactation support is available for these women? Just a few brief comments on this would be helpful.

 Inclusion/Exclusion criteria: what age do you expect most infants to be? Is there a lower limit for inclusion? Breastfed children greater than 12 months old are excluded, but the more significant changes in breast milk PK occur in the first month of life. Drug excretion in colostrum and transitional milk can differ significantly from mature milk. Under “Analysis of primary endpoint” there is mention of transplacental transfer – do you plan to enroll mothers who begin antimalarial treatment while in labor? Recent transplacental transfer could significantly impact infant exposure. While interesting to study, analysis may be difficult if you are including 1) women at all lactation stages (including early lactation) and 2) infants with exposure to AL or DP that started before birth. Please add some comments about how you plan to address different stages of lactation and antimalarial exposure before delivery, or if there are additional exclusions based on recent exposures to antimalarials in pregnancy, and stage of lactation.

**Minor concerns:**

 Abstract, title (and elsewhere):

 Instead of saying “drugs” or “antimalarial drugs”, it would be helpful to specify which ones.

**Introduction:**

 At the end of the second paragraph you mention the 4726 AEs out of 6000 children receiving AL reported by Oluwaseun Egunsola and Kazeem A Oshikoya, which sounds alarming and is worth reporting. However, a comment on the severity and apparent relationship to the study drug might give some context overall (ie. the authors conclude that AL is safe in children). You could also comment on the safety of DP in children.

 In the abstract and introduction you state that “ *drugs are transferred into breast milk in small quantities*” – suggest you amend this to “drugs usually are transferred into breast milk” or “typically” because there are exceptions, which is why you are undertaking this valuable study.

 I found the rationale for the mental health evaluations in the introduction a bit difficult to follow. The concept is presented in paragraph 5 and in detail in paragraph 6, but there is an intervening sentence about the overall PK objectives. I would suggest moving the information about the larger MILK fellowship, followed by some of the details of the questionnaires up to the 5 ^th^ paragraph. This will help the reader place this study in the larger work exploring mental health of postpartum women taking a variety of medications. Paragraph 6 can then be a summary paragraph containing the sentence giving the overview of the PK objectives with a following single sentence reviewing an overview of the mental health objectives.

 The seventh paragraph of the introduction feels more like methods or discussion. Perhaps in the methods it could fit under informed consent or under Publication and dissemination of study results/During protocol development.

**Methods:**

 You mention that dolutegravir-containing ARVs are safe with AL (citation 15). It might be worth also mentioning that there were not significant differences in PK parameters of AL with and without dolutegravir containing ARV in that study, supporting your plan to enroll women regardless of HIV status.

**Objectives:**

 The specific compounds being measured should be clear for each objective and endpoint. Using “antimalarial drugs” here is confusing as you are not planning to measure artemeter or dihydroartemisinin.

**Study design:**

 Recommended PK sampling times for various antimalarials have been published by WWARN and the WHO in 2010 (Basco LK, World Health Organization, Informal consultation organized by the World Health Organization with the technical support of the worldwide antimalarial resistance network. Methods and techniques for assessing exposure to antimalarial drugs in clinical field studies. 2011;162. Available at:

https://www.who.int/docs/default-source/documents/publications/gmp/methods-and-techniques-for-assessing-exposure-to-antimalarial-drugs-in-clinical-field-studies.pdf?sfvrsn=7a2a8c942)

 While the schedule included here is similar to that suggested for AL, the currently proposed schedule is much shorter than that suggested for DP (up to day 63). Though perhaps justifiable as this is included as an exploratory endpoint, it would be good to mention/address this limitation.

 In an area where women are frequently receiving antimalarials, a baseline pre-dose sample seems like it should be required, not optional. Is it possible for the partnering clinics to take this single sample?

**Treatments of participants, drug storage and drug accountability**

 What is known about the storage conditions at these clinics? Is drug quality at these clinics assured (eg. from a reliable national distributer)? While I agree it is good to see the "real life" PKs achieved, considering this will be the first breast milk PK data ever for these drugs, ensuring the drug quality seems important.

**Assessments, Safety:**

 I would recommend recording all AEs. AEs from the sampling procedures, while technically correct, are not particularly meaningful outcomes. Meanwhile, there are no cohorts of prospectively monitored breastfed infants of mothers taking these drugs in the literature. Though the study is not powered for side effects, infant AEs (or the lack thereof) in a monitored environment would add to a data vacuum.

**Analysis of primary endpoint**

**Population pharmacokinetic (pop-PK) analysis:**

 “Population pharmacokinetic modelling will be used to characterize the change in drug and metabolite concentrations across time and estimate parameter characterizing oral absorption of the drug…” parameter should be pleural: parameters.

 “ *Exposure to lumefantrine and piperaquine in breastfed infants will be compared with the range of concentrations achieved in adults given therapeutic doses, assuming a minimal duration of exposure from delivery and a maximal duration from the time the mother started the drug (to account for placental transfer)…*” Are you enrolling women who started their course of AL before delivery? This seems like it would complicate things considerably. PK in pregnant women has been done (with conflicting results). The physiology and drug transfer to offspring in pregnancy and breastfeeding is so different – I would really recommend separating the two time periods or justifying why the decision was made to study combined pregnant/postpartum exposure. (Unless you are referring here to remote previous treatment with AL because of frequent malaria infections – which should also be clarified.)

**End of malaria treatment outcomes:**

 Here it is stated that women will be followed to 30-40 days after treatment, but table 1 indicates end of study will be at day 28 for the majority of women.

 While it is important to study mental health in settings where comprehensive mental health care is not available, there should be some description of what services are available and what kind of referrals will be made in the case of abnormal mental health screenings. What will you do in the case of significant peripartum mood disorders? Suicidality? Peripartum psychosis? There should be some plan to demonstrate the mental health measures are being done ethically (eg. not in an extractive manner).

**Bioanalysis:**

 Developing a breast milk assay can be a major challenge, and it may be necessary to have separate controls for different phases of lactation (eg. colostrum vs mature milk). Is there laboratory support/mentorship from an academic center that has already done this for other drugs?

**Population Pharmacokinetic (pop-PK) analysis:**

 “ *assuming a minimal duration of exposure from delivery and a maximal duration from the time the mother started the drug (to account for placental transfer)*” – Your meaning here is not clear to me. See my comment above. Enrolling mother/infant dyads who have received these drugs before delivery will need to be carefully accounted for in the analysis, especially for infant exposure.

**Quality control and quality assurance:**

 For a complex study of this type, monitoring (internal or external) seems essential, not optional.

**Publication and dissemination of study results:**

 I commend the authors for a well developed plan engaging stakeholders at multiple levels, including, importantly, the community.

**Discussion:**

 Paragraph 4: “ *Secondly, model-based population will…*” do you mean “model based analysis”?

Is the study design appropriate for the research question?

Yes

Is the rationale for, and objectives of, the study clearly described?

Yes

Are sufficient details of the methods provided to allow replication by others?

Partly

Are the datasets clearly presented in a useable and accessible format?

Not applicable

Reviewer Expertise:

malaria, lactation pharmacokinetics, maternal and child health

I confirm that I have read this submission and believe that I have an appropriate level of expertise to confirm that it is of an acceptable scientific standard, however I have significant reservations, as outlined above.

## References

[1] Methods and techniques for assessing exposure to antimalarial drugs in clinical field studies. *World Health Organisation; Worldwide Antimalarial Resistance Network* .10; Reference source

# Response

**Published:** 2023-07-18

**Authors:** RITAH NAKIJOBA

**Affiliations:** Infectious Diseases Institute, Uganda

**Point-by-point response to reviewers’ comments to the** **manuscript,** **“**Pharmacokinetics of drugs used to treat uncomplicated malaria in breastfeeding mother-infant pairs: An observational pharmacokinetic study. We appreciate the thorough review(s) and valuable comments from the editor and the reviewers. We have critically assessed the comments and subsequently revised the manuscript, accordingly, were necessary, while discussing other comments for better clarity, as summarized below.

**Reviewer’s comments Reviewer #2**  " *What is your plan for breast milk sampling e.g. random breast milk sample (not recommended) or complete emptying of both breasts for the duration of the study (recommended by FDA but burdensome and possibly unethical)? Use of a random sample is likely to oversample foremilk, which may have lower drug concentrations due to lower fat content than hind milk, especially considering the lipophilicity of these drugs. Emptying of the fullest breast at the PK timepoints and taking an aliquot of this milk after thorough mixing is a practical option which approximates the dynamics of breastfeeding. (Separate testing of foremilk and hindmilk could be done in selected samples if feasible.)*"

**Response to reviewer’s comments** Breastmilk samples will be obtained pre-dose and at two, four, six, and eight hours after the dose as highlighted on page 4 under the ( **STUDY DESIGN**) section.

 Within the pharmacokinetic sampling windows mothers will be advised to continue breastfeeding their infants normally. Consequently, the proportion fore-to-hind milk is expected to vary across sampling time points. The PK sample taken soon after the infant has suckled will most likely have a higher hind milk fraction compared with that taken long after the infant has suckled. This will most likely translate into a variable influence of fore-to-hind milk fraction on the breast milk drug concentration.

 We agree that the impact of fore-to-hind milk on the measured breast milk concentration needs to be systematically studied. A more robust breast milk sampling methodology that that enables sampling and quantification of drug concentration in fore milk (only) and hind milk (only) needs to be developed and implemented. However, in this current study, we will not be able to implement it.

 While that may be a limitation of our study, it is one we are willing to entertain considering there is hardly any information about these drugs in breastmilk. Also, the FDA-recommended approach of fully emptying both breasts has not been validated in out setting and we have concerns about the feasibility and acceptability of this.

**Reviewer’s comments Reviewer #2 "** *Do you plan to measure the fat content of the milk samples (eg. by creamatocrit) to help explain fluctuations in drug concentrations?*"

**Response to reviewer’s comments **We do not plan to measure the fat content of the milk samples. As explained above, the mothers will be advised to continue breast feeding their infants normally between the pharmacokinetic sampling windows. As such we expect that the proportion of fore-to-hind milk and the creamatocrit will vary depending on the infant suckling patterns. The study will collect 5mls of breastmilk at every PK time point, then the infant will continue breastfeeding as required, test has been revised in the Pharmacokinetic sampling procedures section, page 7 of the manuscript. Women are given health education messages regarding breastfeeding during the study visits.

**Reviewer’s comments Reviewer #2 "** *If women are fully emptying one or both breasts, what will be done with the remaining milk after the aliquot is taken? If it will be fed back to the infant, what measures are in place to minimize disruption of the breastfeeding relationship (eg. will bottles be used, or will the milk be fed by other methods?) What lactation support is available for these women? Just a few brief comments on this would be helpful.*"

**Response to reviewer’s comments **As mentioned above we will not fully empty the breasts, rather breast milk will be sampled at specific time points.

 The mother will continue to breastfeeding their infants normally, text added on page 7 under pharmacokinetic sampling procedures

**Reviewer’s comments Reviewer #2 "** *Inclusion/Exclusion criteria: what age do you expect most infants to be? Is there a lower limit for inclusion? Breastfed children greater than 12 months old are excluded, but the more significant changes in breast milk PK occur in the first month of life. Drug excretion in colostrum and transitional milk can differ significantly from mature milk. Under “Analysis of primary endpoint” there is mention of transplacental transfer – do you plan to enroll mothers who begin antimalarial treatment while in labor? Recent transplacental transfer could significantly impact infant exposure. While interesting to study, analysis may be difficult if you are including 1) women at all lactation stages (including early lactation) and 2) infants with exposure to AL or DP that started before birth. Please add some comments about how you plan to address different stages of lactation and antimalarial exposure before delivery, or if there are additional exclusions based on recent exposures to antimalarials in pregnancy, and stage of lactation.*"

**Response to reviewer’s comments **We do not plan to enroll mothers in labor, we shall enroll mothers who have initiated breastfeeding and have been diagnosed with Malaria. Therefore, it is possible to enroll mothers (and infants who are) a day’s/week postpartum and as such, we have no lower limit of inclusion.

 Indeed, inclusion of mother-infant pairs at a range of postpartum time points will enrich the analysis, and the time after delivery will be an important covariate in the population PK model. Having a small number of mothers who are producing colostrum rather than mature milk would yield important information.

 It is plausible that if a mother took drugs during the days leading up to labor, there could be some residual infant drug exposure either through breast milk or via trans-placental transfer of drugs.

 Predose maternal and infant plasma sample and predose breastmilk samples will be sampled for bioanalysis hence any residual exposure from drug doses taken during pregnancy will be accounted for. Additionally, the post-partum time of pharmacokinetic sampling will be documented and tested as a covariate in the population pharmacokinetic analysis.

 We anticipate that infants that are 12 months and older are taking other foods and are breastfeeding less.

 The case report forms will capture information on past medical history and anti-malaria treatments taken in past weeks.

**Reviewer’s comments Reviewer #2 "** *Minor concerns Abstract tittle “instead of saying “drugs” or “antimalarials” it would be helpful to specify which ones*"

**Response to reviewer’s comments **Thank you for this comment.

 We have updated the manuscript to read “antimalarial drugs” whenever we are referring to these and then “other drugs” when we are not as indicated in the abstract tittle

**Reviewer’s comments Reviewer #2 "** *At the end of the second paragraph you mention the 4726 AEs out of 6000 children receiving AL reported by Oluwaseun Egunsola and Kazeem A Oshikoya, which sounds alarming and is worth reporting. However, a comment on the severity and apparent relationship to the study drug might give some context overall (ie. the authors conclude that AL is safe in children). You could also comment on the safety of DP in children.*"

**Response to reviewer’s comments **Artemether-lumefantrine combination is as safe as ASAQ and DP for use in children, comment is added in the document.

**Reviewer’s comments Reviewer #2 "** *In the abstract and introduction, you state that “drugs are transferred into breast milk in small quantities” – suggest you amend this to “drugs usually are transferred into breast milk” or “typically” because there are exceptions, which is why you are undertaking this valuable study.*"

**Response to reviewer’s comments **Thank you for this suggestion. We have rewritten this sentence as you recommend to read as “drugs usually are transferred into breast milk” on page 1.

**Reviewer’s comments Reviewer #2** " *Found the rationale for the mental health evaluations in the introduction a bit difficult to follow. The concept is presented in paragraph 5 and in detail in paragraph 6, but there is an intervening sentence about the overall PK objectives. I would suggest moving the information about the larger MILK fellowship, followed by some of the details of the questionnaires up to the 5th paragraph. This will help the reader place this study in the larger work exploring mental health of postpartum women taking a variety of medications. Paragraph 6 can then be a summary paragraph containing the sentence giving the overview of the PK objectives with a following single sentence reviewing an overview of the mental health objectives.*"

**Response to reviewer’s comments **Thank you for this suggestion, we have rewritten the introduction section of the protocol to improve clarity as seen on page on page 3.

**Reviewer’s comments Reviewer #2 "** *The seventh paragraph of the introduction feels more like methods or discussion. Perhaps in the methods it could fit under informed consent or under Publication and dissemination of study results/During protocol development"*

**Response to reviewer’s comments **The paragraph has been shifted to publication and dissemination section under protocol development.

**Reviewer’s comments Reviewer #2 "** *Methods You mention that dolutegravir-containing ARVs are safe with AL (citation 15). It might be worth also mentioning that there were not significant differences in PK parameters of AL with and without dolutegravir containing ARV in that study, supporting your plan to enroll women regardless of HIV status.*" 

**Response to reviewer’s comments** Thank you for this helpful comment. We have added the following text “the study will enroll women regardless of their HIV status, basing on previous studies that concluded that were no significant differences in PK parameters of AL with or without dolutegravir containing ARV drugs”, reference 15.

**Reviewer’s comments Reviewer #2 "** *Objectives: The specific compounds being measured should be clear for each objective and endpoint. Using “antimalarial drugs” here is confusing as you are not planning to measure artemether or dihydroartemisinin.*"

**Response to reviewer’s comments** Thank you for this comment. For clarity, we have amended this section to state lumefantrine and desbutyl-lumefantrine

**Reviewer’s comments Reviewer #2 "** *Study design: Recommended PK sampling times for various antimalarials have been published by WWARN and the WHO in 2010 (Basco LK, World Health Organization, Informal consultation organized by the World Health Organization with the technical support of the worldwide antimalarial resistance network. Methods and techniques for assessing exposure to antimalarial drugs in clinical field studies. 2011;162. Available at: https://www.who.int/docs/default-source/documents/publications/gmp/methods-and-techniques-for-assessing-exposure-to-antimalarial-drugs-in-clinical-field-studies.pdf?sfvrsn=7a2a8c942) While the schedule included here is similar to that suggested for AL, the currently proposed schedule is much shorter than that suggested for DP (up to day 63). Though perhaps justifiable as this is included as an exploratory endpoint, it would be good to mention/address this limitation.*"

**Response to reviewer’s comments **Just like the pharmacokinetic sampling schedule for characterisation of lumefantrine pharmacokinetics, the schedule proposed for characterisation of piperaquine pharmacokinetics was evaluated using the stochastic simulation and estimation approach using the model by Tarning et al., 2008 (doi:10.1128/AAC.00955-07), assuming a 70 Kg mother. The proposed protocol still delivered precise parameter estimates and given that piperaquine was being evaluated on an exploratory basis we did not consider sampling further.

 We have added under methods. From the stochastic simulation and estimation evaluation, the proposed sampling schedule for characterisation of piperaquine pharmacokinetics provided precise piperaquine pharmacokinetic parameters estimates. Given that the piperaquine will be evaluated on an exploratory basis, we have not proposed extending the sapling beyond 35 days.

 The PK sampling will be conducted at the IDI clinic rather than other referring sites due to lack of capacity to perform PK procedures.

 It is not really possible to ensure everything is in place at each clinic. However, we will have data on site of recruitment, so any unusually low maternal plasma concentrations could be traced to a particular site.

**Reviewer’s comments Reviewer #2 "** *Treatments of participants, drug storage and drug accountability What is known about the storage conditions at these clinics? Is drug quality at these clinics assured (eg. from a reliable national distributer)? While I agree it is good to see the "real life" PKs achieved, considering this will be the first breast milk PK data ever for these drugs, ensuring the drug quality seems important.*"

**Response to reviewer’s comments **Very true, drug quality is very important; however, the study may not be able to monitor the storage conditions of the different recruitment clinics.

**Reviewer’s comments Reviewer #2 "** *Assessments, Safety: I would recommend recording all AEs. AEs from the sampling procedures, while technically correct, are not particularly meaningful outcomes. Meanwhile, there are no cohorts of prospectively monitored breastfed infants of mothers taking these drugs in the literature. Though the study is not powered for side effects, infant AEs (or the lack thereof) in a monitored environment would add to a data vacuum.*"

**Response to reviewer’s comments** The study will appropriately record and respond to adverse events related to the study procedures as indicated in the Assessment section of the protocol.

**Reviewer’s comments Reviewer #2 **Analysis of primary endpoint Population pharmacokinetic (pop-PK) analysis: “Population pharmacokinetic modelling will be used to characterize the change in drug and metabolite concentrations across time and estimate parameter characterizing oral absorption of the drug…” parameter should be pleural: parameters.

**Response to reviewer’s comments **We agree the parameter should be pleural.

**Reviewer’s comments Reviewer #2 "** *“Exposure to lumefantrine and piperaquine in breastfed infants will be compared with the range of concentrations achieved in adults given therapeutic doses, assuming a minimal duration of exposure from delivery and a maximal duration from the time the mother started the drug (to account for placental transfer) …”  Are you enrolling women who started their course of AL before delivery? This seems like it would complicate things considerably. PK in pregnant women has been done (with conflicting results). The physiology and drug transfer to offspring in pregnancy and breastfeeding is so different – I would really recommend separating the two time periods or justifying why the decision was made to study combined pregnant/postpartum exposure. (Unless you are referring here to remote previous treatment with AL because of frequent malaria infections – which should also be clarified.)"*

**Response to reviewer’s comments **We will not enroll women who start AL treatment during/ prior labour, the study shall recruit outpatient breastfeeding women diagnosed with uncomplicated malaria.

 Because of the logistics involved in conducting Intensive PK on the wards, women that are admitted and are in a labor will not be enrolled in the study.

**Reviewer’s comments Reviewer #2 "** *End of malaria treatment outcomes: Here it is stated that women will be followed to 30-40 days after treatment, but table 1 indicates end of study will be at day 28 for the majority of women.   While it is important to study mental health in settings where comprehensive mental health care is not available, there should be some description of what services are available and what kind of referrals will be made in the case of abnormal mental health screenings. What will you do in the case of significant peripartum mood disorders? Suicidality? Peripartum psychosis? There should be some plan to demonstrate the mental health measures are being done ethically (eg. not in an extractive manner).*"

**Response to reviewer’s comments **We have clarified this statement to say that participants will be followed up to day 28 if on AL and up to day 35 if on DP. Table 1 indicates that participants on DP will be followed up for 35 days.

 Participants with abnormal mental health screenings will be referred to the institution mental health clinic for further review, text added in PK sampling procedures section.

 The Infectious Diseases institute (IDI) has a mental health clinic that runs on Thursday every week, suspected cases will be referred to the clinic Psychiatrist.

 Regardless of any complication, the clinic has specialists that are consulted.

**Reviewer’s comments Reviewer #2 "** *Bioanalysis: Developing a breast milk assay can be a major challenge, and it may be necessary to have separate controls for different phases of lactation (eg. colostrum vs mature milk). Is there laboratory support/mentorship from an academic center that has already done this for other drugs?*"

**Response to reviewer’s comments **The study has a collaboration between IDI, University of Liverpool and University of Cape Town, and that in both partner sites there are GCLP accredited laboratories. Liverpool has significant strength in antiretroviral drugs, and UCT in antituberculotic drugs.

 The lab has an EQA system with the Netherlands laboratory, and the study has a collaborative partnership with the UCT laboratory whereby the laboratory staff receive regular mentorship and technical guidance remotely.

 Also, the IDI lab team have strong working relationships with the lab teams at both of these partner organizations. This text has been included on page 9 of the PDF version.

**Reviewer’s comments Reviewer #2 "** *Population Pharmacokinetic (pop-PK) analysis: “assuming a minimal duration of exposure from delivery and a maximal duration from the time the mother started the drug (to account for placental transfer)” – Your meaning here is not clear to me. See my comment above. Enrolling mother/infant dyads who have received these drugs before delivery will need to be carefully accounted for in the analysis, especially for infant exposure."*

**Response to reviewer’s comments **Thank you for this comment, because of the logistics involved in conducting Intensive PK on the wards, women that are admitted and are in a labor will not be enrolled in the study.

**Reviewer’s comments Reviewer #2 "** *Quality control and quality assurance: For a complex study of this type, monitoring (internal or external) seems essential, not optional.*"

**Response to reviewer’s comments **This study will be monitored internally because participants will take the standard of care drugs prescribed from the respective clinics, no changes will be made in the administered medication as written in the treatments of participants, drug storage and drug Accountability section of the protocol. Also, the IDI internal monitoring SOPs and the structures of Research Office guide the study to maintain effective quality control practices.

**Reviewer’s comments Reviewer #2 "** *Publication and dissemination of study results: I commend the authors for a well-developed plan engaging stakeholder at multiple levels, including, importantly, the community.*"

**Response to reviewer’s comments **Thank you for the positive feedback. We will also report back on the public engagement portfolio.

**Reviewer’s comments Reviewer #2 "** *Discussion: Paragraph 4: “Secondly, model-based population will…” do you mean “model-based analysis”?*"

**Response to reviewer’s comments **model-based population revised to model-based analysis on page 11.

# Reviewer report: Reviewer response for version 1

**DOI:** 10.21956/wellcomeopenres.20528.r54024

**Published:** 2023-02-14

**Authors:** Andre Daher

**Affiliations:** 1 Oswaldo Cruz Foundation, Rio de Janeiro, Brazil

The study protocol “Pharmacokinetics of drugs used to treat uncomplicated malaria in breastfeeding mother-infant pairs: An observational pharmacokinetic study” address an important issue. Breastfeeding mothers are not usually included in clinical trials and their prescriptions are mostly off-label. There is a lack of reliable evidence and the study population has been neglected. The protocol also describes a careful approach that includes the mother’s mental health and children's well-being assessments.

 However, a few major study's design weaknesses should be revised.

- Although the study protocol put emphasis on Dihydroartemisinin-piperaquine pharmacokinetic evaluations, piperaquine is not included in any endpoint. In the study design section, the authors justify that it is not the first-line treatment in Uganda. The piperaquine PK parameters will be an exploratory endpoint, and this should be clearly stated in the protocol. Collecting enough data to feed a model is unlike. Moreover, even though the sample size has not been calculated for any comparison, having subsets of patients may lose power to meaningful lumefantrine PK results. The sample size may be calculated on the expected coefficient of variation lumefantrine PK parameters. Expected losses of follow-up and a number of dihydroartemisinin-piperaquine users should be added to that sample size.
- Similarly, the inclusion of HIV-positive and antiretroviral users might impact the results. Although this population should not be excluded, and “ *data on concomitant medication will be recorded and included as a covariate in the pharmacokinetic analysis*” the drug-drug interaction (DDI) and immunological status (that should be recorded and included as a covariate as well) might influence the overall sample size. The DDI is an additional challenge to the analytical team, as these drugs may need to be taken into account during the analytical methodology development. 
- The second major comment is regarding the infant blood sample collection. If the parent ACT drug concentration is very low or even below the low limit of quantification in breast milk (page 10), there is no rational to collect infant blood samples. Exposing infants to study procedures and their risks (even if minimal) should have a strong rationale. Additionally, it will adversely bias the children's well-being assessments. One possible approach would be splitting the study into two tiers. A pilot cohort to evaluate the drug concentration only in the breast milk of up to six mothers may make the study design more robust.
- The PK sample schedule could be further justified. The protocol states that: “ *Due to the long plasma half-life of lumefantrine (approximately six days), sampling will be performed up to day 14 to characterize the terminal elimination of the drug.*” Twice the time of the plasma half-life might not be enough to characterize the terminal elimination of the drug. As a rough parameter, the regulatory bioequivalence guidance recommends 5 half-lives duration to PK sample schedule. This is also true for the planned piperaquine and desbutyl-lumefantrine PK sample schedule.
- Further details on infant PK sampling schedule should also be provided as their drug blood concentrations might be influenced by their mother's drug blood concentrations (page 8), but also by the time since their last breastfeeding.
- The second exclusion criterion is “ *Breastfed infant is aged over 12 months. Time-dependent changes in the quality of breast milk and infant feeding practices may affect the mother-to-infant transfer of drugs*”. Please consider that the same rational should be applied to newborns and colostrum.

**Minor comments:**

- Please revise the study design text as it does not reflect figure 1 accurately. It is relevant to inform when hospital/clinics admission occurs.
- Although this is an observational study and “ *All women (and infants) will continue to receive their malaria treatment as prescribed by the physician where they receive clinical care*” it is a very important to record and control for drug manufacturer and treatment adherence as covariates in the analyses. Drug formulation does impact PK.
- Even if “ *there is no formal requirement for notification of adverse events to the regulatory authorities*” IRB should be informed of adverse events in a timely fashion.
- The “ *validation of breastmilk assay/ donor breastmilk*” section states that “blank” breast milk is likely to be donated by staff and students working within IDI. Please notice that study centre staff and students should be considered a vulnerable population, as they might feel pressured to donate. Non-study-related donors are preferable and informed consent should be provided.
- Data management quality procedures, such as data double entry, and use of open source auditable e-CRF (as RedCap) should be encouraged and described in the protocol. Planning to share the data on platforms such as WWARN also helps to reinforce the importance of data quality, and makes the study more relevant to the scientific community.

Finally, my congratulations to the authors to plan such a difficult study on a neglected population.

Is the study design appropriate for the research question?

Yes

Is the rationale for, and objectives of, the study clearly described?

Yes

Are sufficient details of the methods provided to allow replication by others?

Yes

Are the datasets clearly presented in a useable and accessible format?

Not applicable

Reviewer Expertise:

Clinical research, drugs, Malaria, NTD.

I confirm that I have read this submission and believe that I have an appropriate level of expertise to confirm that it is of an acceptable scientific standard, however I have significant reservations, as outlined above.

# Response

**Published:** 2023-07-18

**Authors:** RITAH NAKIJOBA

**Affiliations:** Infectious Diseases Institute, Uganda

**Point-by-point response to reviewers’ comments to the manuscript,** **“** *Pharmacokinetics of drugs used to treat uncomplicated malaria in breastfeeding mother-infant pairs: An observational pharmacokinetic study."* We appreciate the thorough review(s) and valuable comments from the editor and the reviewers. We have critically assessed the comments and subsequently revised the manuscript, accordingly, were necessary, while discussing other comments for better clarity, as summarized below.

**Reviewer’s comments -** **Reviewer #1** " *Although the study protocol put emphasis on Dihydroartemisinin-piperaquine pharmacokinetic evaluations, piperaquine is not included in any endpoint. In the study design section, the authors justify that it is not the first-line treatment in Uganda. The piperaquine PK parameters will be an exploratory endpoint, and this should be clearly stated in the protocol.    sample size may be calculated on the expected coefficient of variation lumefantrine PK parameters. Expected losses of follow-up and a number of dihydroartemisinin-piperaquine users should be added to that sample size.*"

**Response to reviewer’s comments **If a breastfeeding mother receiving dihydroartemisinin-piperaquine is identified, she shall be invited for intensive pharmacokinetic sampling as an exploratory endpoint, this was stated on page 4 of the manuscript.

 Thank you for this comment, while we agree that loss to follow-up should be considered when deciding on a sample size, in this instance, our sample size calculations did not. For Pharmacokinetic studies, it is not only the number of individuals to consider but also the number of samples per individual.  Antimalarial treatment is short, (3 days) only. Therefore, we expect that most mothers will be adherent and remain in the study. Secondly, the PK modelling technique that we will use to analyze these samples is more forgiving of loss to follow up than more traditional methods like non-compartmental analysis. Since the PK samples are analyzed at a population level, any samples collected from an individual who is then lost to follow up can still be included in the study since they provide information.

**Reviewer’s comments -** **Reviewer #1**  " *Similarly, the inclusion of HIV-positive and antiretroviral users might impact the results. Although this population should not be excluded, and “data on concomitant medication will be recorded and included as a covariate in the pharmacokinetic analysis” the drug-drug interaction (DDI) and immunological status (that should be recorded and included as a covariate as well) might influence the overall sample size. The DDI is an additional challenge to the analytical team, as these drugs may need to be considered during the analytical methodology development.*"

**Response to reviewer’s comments** Under the INTRODUTION – PROTOCOL section, we stated that data on concomitant medication will be recorded and included as a covariate in the pharmacokinetic analysis. We acknowledge that sample size would influence ability to estimate the Drug’s DDI potential, however this study will only evaluate the DDI potential exploratively rather than perform a formal evaluation that requires sample size calculation.

 The interim analysis will guide us on the prevalence of Coadministered drugs with DDI potential and highlight the possible implications on the development of the analytical methodology.

**Reviewer’s comments -** **Reviewer #1** " *The second major comment is regarding the infant blood sample collection. If the parent ACT drug concentration is very low or even below the low limit of quantification in breast milk (page 10), there is no rational to collect infant blood samples. Exposing infants to study procedures and their risks (even if minimal) should have a strong rationale. Additionally, it will adversely bias the children's well-being assessments. One possible approach would be splitting the study into two tiers. A pilot cohort to evaluate the drug concentration only in the breast milk of up to six mothers may make the study design more robust.*"

**Response to reviewer’s comments **You raise an important issue concerning the wellbeing of children and not exposing them to study procedures needlessly. However, we have done similar studies in breastfeeding mother infant pairs and that it has not been associated with any harms/ reported risks.

 Also, we will do an interim analysis midway the study after about 6-7 women to see what the concentrations of drug in breastmilk are. This will give us an idea on whether we should continue sampling the infants or not.

**Reviewer’s comments -** **Reviewer #1** " *The PK sample schedule could be further justified. The protocol states that: “Due to the long plasma half-life of lumefantrine (approximately six days), sampling will be performed up to day 14 to characterize the terminal elimination of the drug.” Twice the time of the plasma half-life might not be enough to characterize the terminal elimination of the drug. As a rough parameter, the regulatory bioequivalence guidance recommends 5 half-lives duration to PK sample schedule. This is also true for the planned piperaquine and desbutyl-lumefantrine PK sample schedule.*" 

**Response to reviewer’s comments **Our decision to sample up to for lumefantrine PK up to day 14 is provided under **“Data analysis/statistical methods, Sample size determination.”**

 Using the stochastic simulation and estimation (SSE) approach based on the model by Hodel EMS *et al*., 2013, the sampling schedule proposed would yield precise parameter estimates for an average (70 Kg) mother. Additionally, the average lumefantrine concentration across time from the SSE showed that by day 8 after treatment the plasma concentration of lumefantrine would already be below 200 ng/mL (the Lower Limit of Quantitation for the validated lumefantrine assay). We included days 8, 12 and 14 as caution to account for potential skewness in model prediction for lower populations given our study population.

**Reviewer’s comments -** **Reviewer #1** Further details on infant PK sampling schedule should also be provided as their drug blood concentrations might be influenced by their mother's drug blood concentrations (page 8), but also by the time since their last breastfeeding.

**Response to reviewer’s comments **In the study design section infant sampling would be performed at maternal trough as well as at a random time point over the eight-hour pharmacokinetic sampling visit. It is true that several factors, including breast milk concentrations, infant feeding habits and infant pharmacokinetic attributes affect the resulting infant concentrations.

**Reviewer’s comments -** **Reviewer #1 **The second exclusion criterion is “Breastfed infant is aged over 12 months. Time-dependent changes in the quality of breast milk and infant feeding practices may affect the mother-to-infant transfer of drugs”. Please consider that the same rational should be applied to newborns and colostrum.

**Response to reviewer’s comments** One of the key covariates of the population pharmacokinetic model is the post-natal age. This variable would account for the impact of time-dependent factors such as composition of breast milk and breast milk intake on infant concentrations. Also, the 12 months cut off was a pragmatic choice. At 12 months, majority of infants have been weaned fully and breastmilk is given more as a supplement and not the main source of sustenance for the infant. 

**Reviewer’s comments -** **Reviewer #1 **Please revise the study design text as it does not reflect figure 1 accurately. It is relevant to inform when hospital/clinics admission occurs.

**Response to reviewer’s comments** The clinic admissions will occur on day 1 of Malaria treatment, this information is included on page 4 under the study design section.

**Reviewer’s comments -** **Reviewer #1**  Although this is an observational study and “All women (and infants) will continue to receive their malaria treatment as prescribed by the physician where they receive clinical care” it is a very important to record and control for drug manufacturer and treatment adherence as covariates in the analyses. Drug formulation does impact PK. 

**Response to reviewer’s comments** We agree, drug manufacturers/different brands can indeed influence the PK of a drug. We intend to enroll women attending government aided facilities (KCCA clinics). Since these facilities are supplied with drug by the government drug agency. We expect that the brands used across all the facilities are ideally the same. Never the less, we will capture the details of treatment (including brand and manufacturer) on the concomitant medication Case Report Form.

 Also, patients are being provided with “adherence cards” to state the time and date of each dose. And since the malaria treatment is only 3 days, we don’t anticipate adherence to be a problem.

**Reviewer’s comments -** **Reviewer #1 **Even if “there is no formal requirement for notification of adverse events to the regulatory authorities” IRB should be informed of adverse events in a timely fashion

**Response to reviewer’s comments **The adverse events will be captured on the adverse event CRF and serious adverse events will be notified to relevant regulatory authorities.

**Reviewer’s comments -** **Reviewer #1 **The “ *validation of breastmilk assay/ donor breastmilk*” section states that “blank” breast milk is likely to be donated by staff and students working within IDI. Please notice that study centre staff and students should be considered a vulnerable population, as they might feel pressured to donate. Non-study-related donors are preferable and informed consent should be provided.

**Response to reviewer’s comments** It is an interesting tension - we felt that staff and students would understand the study objectives, and we had drawn from similar practices that are used for blank blood donation in other centres (i.e.The University of Liverpool). We do have informed consent for donating breast milk.

 The non-study related donors have been approached to donate breastmilk.

**Reviewer’s comments -** **Reviewer #1** Data management quality procedures, such as data double entry, and use of open-source auditable e-CRF (as RedCap) should be encouraged and described in the protocol. Planning to share the data on platforms such as WWARN also helps to reinforce the importance of data quality, and makes the study more relevant to the scientific community.

**Response to reviewer’s comments** Indeed, study data will be collected using Research electronic data capture (REDCAP). More details are provided in the study data management plan.

 Raw anonymized data might be shared on the Zenodo online repository
